# Foreign Aid in Transition: A Critical Assessment of USAID's Withdrawal From Five Countries in Sub‐Saharan Africa

**DOI:** 10.1002/puh2.70368

**Published:** 2026-10-27

**Authors:** Ketan Tamirisa, Faraan O. Rahim, Sarah Ali, Alaa Shilleh, Anzibert Rugakingira, Philip Bakahirwa, Michée Kanda

**Affiliations:** ^1^ Washington University in St. Louis St. Louis Missouri USA; ^2^ Harvard Medical School Boston Massachusetts USA; ^3^ University of Pittsburgh Pittsburgh Pennsylvania USA; ^4^ University of La Verne La Verne California USA; ^5^ Ministry of Health of Tanzania Dodoma Tanzania; ^6^ Ministry of Health of Uganda Kampala Uganda; ^7^ Ministry of Health of the Democratic Republic of the Congo Kinshasa Democratic Republic of the Congo

**Keywords:** africa, global health, health system

## Abstract

In January 2025, a US executive order initiated a significant restructuring of USAID, halting nearly all international spending for 90 days and terminating a substantial portion of its foreign aid contracts. This policy shift threatens to unravel decades of development progress, particularly in sub‐Saharan Africa, where USAID has been integral to health, food security, governance, and economic development. This commentary develops a multi‐phase transition framework to address the dissolution's impact on five key beneficiary countries: Tanzania, Kenya, Rwanda, the Democratic Republic of the Congo (DRC), and Ethiopia. These nations face setbacks in maternal and child health, disease prevention, agricultural productivity, and governance. We propose a structured approach to resilience, emphasizing diversified funding sources, progressive tax reforms, enhanced domestic resource mobilization, public‐private partnerships, and regional cooperation. By adopting these strategies, sub‐Saharan African nations can mitigate the effects of USAID's absence, safeguard vital development progress, and transition toward greater self‐reliance amid shifting global aid dynamics.

## 1 Introduction

In January 2025, President Donald Trump signed an executive order placing nearly all US international spending on pause for 90 days [1] and terminating 90% of foreign aid contracts for the United States Agency for International Development (USAID). USAID began implementing a reduction‐in‐force affecting approximately 1600 personnel stationed in the United States [2] and has been formally dissolved as an independent agency. This abrupt dissolution threatens decades of development and humanitarian progress.

Over the past six decades, USAID has played a critical role in global health, economic development, disaster relief, and humanitarian assistance [3]. In 2024 alone, USAID provided over $6.5 billion in humanitarian assistance across 47 sub‐Saharan African countries. In this commentary, we examine the impact of USAID's dissolution on five sub‐Saharan African countries that have historically been significant beneficiaries of USAID support: Tanzania, Kenya, Rwanda, the Democratic Republic of Congo (DRC), and Ethiopia. Between 2001 and 2024, Ethiopia ($16.69 billion), Kenya ($9.75 billion), and the DRC ($8.8 billion) were the largest sub‐Saharan African recipients of USAID funding. Through programs such as PEPFAR and “Feed the Future,” USAID has helped mitigate rising food insecurity across these regions while supporting agricultural improvement, particularly in Tanzania, Kenya, and Rwanda. In addition, USAID has played a critical role in strengthening medical infrastructure and promoting disease prevention efforts, including malaria, HIV/AIDs, and COVID‐19. USAID support has also been essential in bolstering economic infrastructure and governance systems, particularly in Kenya, the DRC, and Ethiopia.

## 2 Tanzania

Despite steadily increasing economic growth in recent years, Tanzania's industrial productivity has dropped nearly 20% over the past three decades, and the country remains vulnerable to regional conflicts, climate shocks, and a narrow tax base [4]. USAID's 2023 “Feed the Future” program has been pivotal in improving agricultural productivity and promoting food security. Its targeted health initiatives, such as PEPFAR, have reduced child and AIDS‐related mortality. Environmental conservation initiatives, such as the Jane Goodall Institute's TACARE, have helped promote biodiversity preservation and food sustainability [5]. Without USAID, food insecurity may rise, reversing progress in reducing malnutrition. Healthcare services could deteriorate, limiting access to essential disease prevention and treatment programs. Conservation efforts may stall, increasing deforestation and habitat loss. In addition, weakened institutional capacity could hinder governance and development efforts.

## 3 Kenya

Still in the process of recovering from the COVID‐19 pandemic, Kenya faces a 40% poverty rate, climate change, internal unrest, gender‐based violence, and inadequate healthcare access. USAID has been a cornerstone in bolstering Kenya's healthcare, food security, and economic systems, contributing an average of $931 million annually between 2010 and 2018 [6]. Programs like Jacaranda Health and “Feed the Future” have also advanced maternal health and climate‐resilient agriculture. USAID has also strengthened anti‐corruption efforts, digital financial services, and infrastructure to promote economic stability and align with Kenya's “Vision 2030” goals [7]. The dissolution of USAID poses significant threats to Kenya's health, food security, and governance. HIV/AIDS programs face potential collapse, and funding gaps could undermine maternal health and disease control efforts. In agriculture, decreased support for farmers might lead to rising food prices and increased malnutrition. Governance efforts could also weaken, undermining anti‐corruption reforms and slowing infrastructure growth, which may discourage investment.

## 4 Rwanda

Despite economic progress and stability, Rwanda faces human rights challenges, political instability, and high food insecurity [8, 9]. USAID programs like the Ireme health system and the Akazi Kanoze Youth Livelihood Project have improved employment outcomes, enhanced livelihoods, and boosted the economy [10]. With USAID's assistance, Rwanda saw an 18.5% reduction in the population living below the poverty line from 2005 to 2016 [11]. In addition, USAID's malaria mitigation program, which provided mosquito nets and insecticide spraying, led to a reduction in malaria incidence by up to 70% in some rural areas. Moreover, the USAID Global Health Supply Chain Program has supplied Rwanda with 32% of its COVID‐19 vaccine doses [12]. USAID's dissolution threatens health commodity supply chains, youth employment efforts, and agricultural advancements. Without USAID support, pharmaceutical/vaccine access may falter, and food insecurity challenges could rise, reversing development gains.

## 5 Democratic Republic of the Congo

The DRC is plagued by violence from multiple armed groups, especially from the M23 rebel group in the eastern region [13]. Over 7 million people face internal displacement, with women and children particularly affected. Limited access to health services and low vaccination rates increase disease outbreak risks. USAID, as the largest donor to the country's health sector, protected over 11 million people in 2024 from malaria, tuberculosis, and AIDS [14]. The agency's programs have improved maternal and child health services, provided potable water, supported internally displaced populations, and offered immediate food security programs. USAID's dissolution could lead to increased malnutrition, reduced access to clean water, and collapse of essential medical infrastructure. Furthermore, the withdrawal of US humanitarian aid may create a power vacuum. This is particularly concerning in the mineral‐rich eastern DRC, where control over resources like coltan—critical for global technologies—has long been contested [13]. Without USAID, rival international actors from Rwanda and beyond may expand their influence, potentially exacerbating conflict.

## 6 Ethiopia

Ethiopia is still recovering from the 2‐year Tigray conflict, and faces ethnic tensions, widespread poverty, healthcare inaccessibility, and climate‐induced food shortages. Since 2020, USAID has invested approximately $3.6 billion in aid in Ethiopia, addressing both conflict and climate‐induced crises [15]. In addition, USAID's “Feed the Future” and PEPFAR programs have helped reduce food insecurity and significantly reduced disease burdens. USAID's dissolution severely threatens Ethiopia's goal of becoming a prosperous, middle‐income country by 2025 [15]. Funding cuts could worsen the food crisis, deepening food, healthcare, and economic instability for millions in need [16].

## 7 Empowering Sub‐Saharan Africa Beyond USAID

While the absence of USAID's support presents significant challenges, Tanzania, Kenya, Rwanda, the DRC, and Ethiopia can take targeted steps to safeguard governance, public health, security, and economic development. These nations must identify alternative funding sources while transitioning toward locally owned and sustainable health and development systems.

Before new financing mechanisms are introduced, these governments must establish clear ownership over existing USAID‐supported programs, particularly in healthcare. Through asset mapping, governments can determine which programs, such as HIV/AIDS treatment under PEPFAR and primary care delivery, can be absorbed by local Ministries of Health and which require continued partnerships with private providers or nonprofit organizations. In addition, institutional capacity building must enable local organizations and government agencies to transition from implementing donor‐directed projects to independently managing these programs. This would strengthen financial management, procurement systems, and technical expertise within these nations. Without this shift, new financing mechanisms may reinforce dependency under new donors rather than promoting sustainable, locally owned systems. While prioritizing local ownership may slow short‐term implementation, it will enhance long‐term sustainability of these systems and mitigate the risk of collapse in the event of donor withdrawal.

Following ownership consolidation, in the next phase, these countries must adopt a diverse financial model in which multiple forms of capital are leveraged in tandem. Concessional financing from multilateral institutions such as the World Bank [17], African Development Bank (AfDB) [18], and the International Monetary Fund (IMF) [19] can support large‐scale investments in health infrastructure. However, these financing mechanisms introduce important trade‐offs. Recent geopolitical instability and global economic shocks have constrained the flexibility of multilateral financing mechanisms. Rising sovereign debt burdens, higher global interest rates, and shifting donor priorities have reduced concessional lending capacity and slowed disbursement timelines, particularly for low‐income countries. As a result, reliance on IMF and AfDB financing requires careful consideration of debt sustainability, loan conditionality, and the risk of pro‐cyclical fiscal tightening that may undermine health system resilience [20].

At the same time, utilizing domestic capital by strengthening domestic resource mobilization is essential for sustaining core health services and reinforcing national ownership. To do so, these sub‐Saharan African countries must strengthen domestic tax collection systems. This will help improve revenue collection and facilitate fiscal management. Implementing progressive tax reforms can ensure that wealthier individuals and corporations contribute more effectively. In addition, strengthening value‐added tax (VAT) compliance can further drive government revenue higher. Sub‐Saharan African governments should enhance regulations to combat tax evasion and illicit financial flows. This is especially important for countries like the DRC, which has vast mineral wealth and therefore must strengthen regulatory oversight of mining revenues to prevent tax losses from illegal mineral exports [21]. Similarly, Kenya should reinforce anti‐corruption measures to ensure that tax revenues are properly allocated and utilized. Rwanda and Kenya, both leaders in digital innovation, can work to expand digital tax systems to improve tax compliance and efficiency, ensuring that online businesses and mobile money transactions contribute to national revenue sources [22]. While domestic capital offers the highest level of sustainability, it remains constrained in low‐income settings and is subject to political and economic volatility.

Philanthropic organizations such as the Bill and Melinda Gates Foundation [23], Gavi, the Vaccine Alliance [24], the Global Fund to Fight AIDS [25], and the Wellcome Trust [26] can provide flexible and rapid funding to support innovation and pilot programs. However, such funding is often short term and donor‐driven, which would require alignment with national health strategies to ensure sustainability, thereby promoting longer‐term investment in disease prevention and treatment rather than short‐term, project‐based funding.

Leveraging public‐private partnerships to accelerate development across sub‐Saharan Africa is essential. To drive improvements in healthcare, Kenya's Ministry of Health can collaborate with private hospitals and pharmaceutical firms to sustain HIV/AIDS and maternal health programs. Ethiopia and Rwanda should expand local pharmaceutical production to reduce reliance on imported medicines. To combat agricultural losses and decrease the overall poverty rate, Tanzania and Ethiopia can partner with agribusiness firms like OCP Africa [27] and Alliance for a Green Revolution in Africa (AGRA) [28] to boost agricultural productivity, focusing on drought‐resistant crops and advanced irrigation systems. Renewable energy initiatives must also be instituted. The DRC and Tanzania, both rich in hydro and solar energy potential, should attract foreign direct investment (FDI) from energy companies to expand off‐grid solar solutions and improve rural electrification [4, 29]. Kenya and Rwanda can also integrate private sector investment in fintech and broadband expansion to enhance access to financial services and education, especially for youth. Similarly, partnerships in the education sector like the Vodacom e‐School initiative in South Africa must be sustained [30]. Private organizations like Aga Khan and Vodacom should continue working with government school systems to address funding shortages and expand digital learning opportunities [31]. While private‐sector engagement can drive efficiency and innovation, it introduces equity concerns and must be supported by strong regulatory frameworks to ensure affordability and access for vulnerable populations.

In addition, bilateral agreements with China, the European Union (EU), the Gulf States, and Japan can be leveraged to further diversify financing sources and support sector‐specific investments in agriculture, infrastructure, and pharmaceutical manufacturing. For instance, Kenya could expand its collaboration with the EU's Global Gateway initiative to enhance digital infrastructure and renewable energy projects [20]. Similarly, Ethiopia can bolster trade and aid ties with Turkey and India to support local pharmaceutical manufacturing and agricultural mechanization, reducing dependence on imported medicines and food supplies. However, geopolitical considerations and shifting donor priorities may affect reliability and strategic alignment of such partnerships.

In the final phase, financing transitions must be embedded within durable institutional and regional systems that prioritize continuity of service delivery to ensure that the developments and programs achieved under USAID are sustained. Blended finance systems, including pooled funding arrangements, combine public, philanthropic, and private funding to support essential services. In this model, philanthropic funding can absorb some financial risk, encouraging private‐sector investment, while governments use these pooled resources to sustain service delivery. Policy alignment is also critical. National development and health strategies must transition donor‐driven reporting frameworks to domestically accountable systems that prioritize population health outcomes and long‐term system resilience. This shift is essential in maintaining consistency in program implementation as funding sources evolve.

Regional cooperation with neighboring countries is essential in institutionalizing continuity. Countries in sub‐Saharan Africa must strengthen collaboration through regional trade, security, and infrastructure projects. Tanzania and Kenya should leverage the African Continental Free Trade Area (AfCFTA) agreements to increase cross‐border trade in agricultural products and manufactured goods, reducing dependence on external markets and boosting economic resilience [32]. Ethiopia and Kenya should work with local and national organizations to maintain disease surveillance and strengthen vaccine distribution networks across East Africa through targeted investments in cold‐chain infrastructure, digital logistics management systems, and workforce training for community‐based health workers. Investment in independent regional health institutions and regional vaccine production hubs can also help ensure that health infrastructure is self‐sustainable across these nations and resilient during geopolitical and financial challenges. In addition, strengthening surveillance capacity requires integrated disease reporting platforms, sustained financing for national public health institutes, and regional laboratory partnerships, assuming sufficient governance capacity and political commitment to support long‐term system maintenance despite funding shortages. The DRC and Rwanda must engage with the African Union (AU) and the East African Community (EAC) to destabilize armed groups in eastern DRC [33, 34]. Both Tanzania and Rwanda should expand cross‐border transport and electricity‐sharing initiatives to enhance economic resilience and energy security. The success of these alternative financing and governance strategies ultimately depends on political stability, institutional capacity, and sustained domestic budgetary commitment, underscoring the need for realistic implementation timelines and context‐specific policy design.

## Author Contributions

**Ketan Tamirisa**: writing – original draft, writing – review and editing, conceptualization, investigation, validation, methodology, visualization. **Faraan O. Rahim**: investigation, conceptualization, methodology, validation, visualization, writing – review and editing. **Sarah Ali**: conceptualization, investigation, visualization, validation, methodology, writing – review and editing. **Alaa Shilleh**: conceptualization, investigation, writing – review and editing, visualization, validation, methodology. **Anzibert Rugakingira**: writing – review and editing, visualization, validation, methodology, conceptualization, and investigation. **Philip Bakahirwa**: writing – review and editing, supervision. **Michée Kanda**: writing – review and editing, supervision.

## Funding

The authors have nothing to report.

## Conflicts of Interest

The authors declare no conflicts of interest.

## Data Availability

Data sharing is not applicable to this article, as no datasets were generated or analyzed during the current study.
